# Dorsal Translocation of the Flexor Pollicis Longus Tendon Following Pediatric Both Bone Forearm Fracture

**DOI:** 10.1016/j.jhsg.2024.06.007

**Published:** 2024-07-15

**Authors:** Jenny Stephanie Ventura, Karlos Manzanarez Felix, Joshua Taylor Lackey, Amber Rachel Leis

**Affiliations:** ∗ University of California, Irvine School of Medicine, Irvine, CA; † Department of Orthopedic Surgery, University of California, Irvine, CA; ‡ Department of Plastic Surgery, University of California, Irvine, CA; § Department of Plastic Surgery, Children's Hospital of Orange County, Orange, CA

**Keywords:** Flexor pollicis longus, Pediatrics, Radius and ulnar shaft fracture, Tendon entrapment

## Abstract

Tendon entrapment is a rare complication of closed forearm fractures. A 16-year-old boy sustained a type 1 open both bone forearm fracture after falling from a skateboard. The injury was initially managed with irrigation, debridement, and flexible intramedullary nailing. Seven weeks after surgery, a flexion contracture of the ipsilateral thumb interphalangeal joint was noted. Subsequent hardware removal and hand therapy failed to improve thumb extension. The patient was taken to the operating room for planned tenolysis and possible tendon reconstruction. Intraoperatively, the flexor pollicis longus tendon was found to be wrapped around the radial shaft as an apparent complication of the initial procedure, which necessitated division and reconstruction of the tendon. To our knowledge, this is the first pediatric reported case of dorsal flexor pollicis longus tendon entrapment through the fracture site in a both bone forearm fracture requiring tendon reconstruction. This case highlights a unique surgical approach to a novel complication of pediatric both bone forearm fracture.

Both bone forearm fractures (BBFFs) comprise approximately 50% of pediatric fractures.^1^ Typically, pediatric BBFFs are treated with a closed reduction and casting. Surgical management is reserved for cases with significantly displaced or angulated fractures. Flexor or extensor tendon entrapment is a rare complication of both closed and open treatment of pediatric BBFFs.^1^ The middle and ring finger flexor digitorum profundus (FDP) tendons are the most commonly entrapped, with a single documented case involving the index FDP and flexor pollicis longus (FPL). Surgical management typically consisted of myotenolysis.^2^ We present a unique case of a 16-year-old patient with FPL entrapment leading to dorsal displacement of the tendon around the radius after healing of a BBFF treated with flexible intramedullary fixation requiring secondary tendon reconstruction.

## Case Report

**Figure 1:**
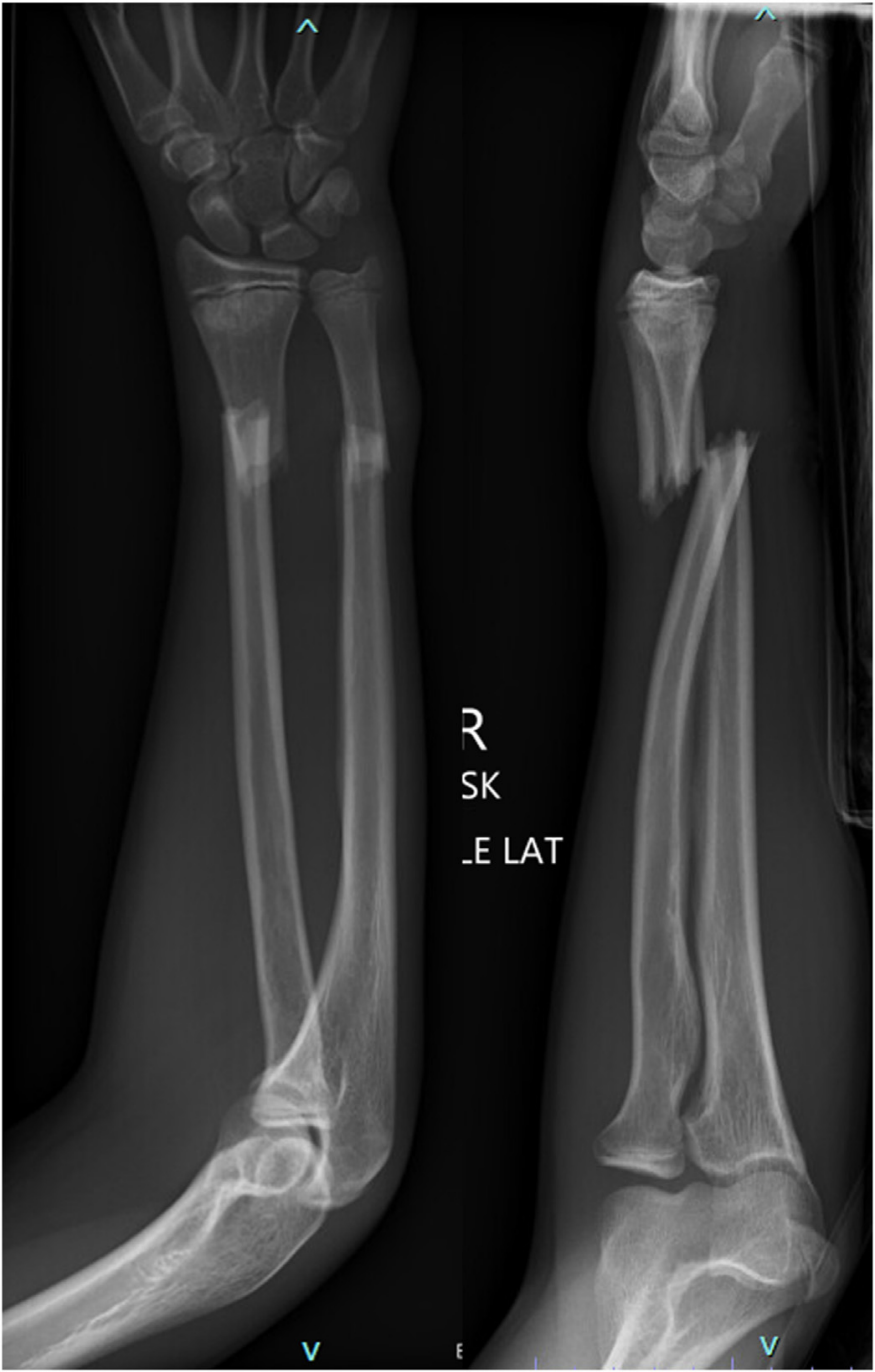
Lateral and poster–anterior radiographs at the time of injury demonstrating a distal one-third radial and ulnar shaft fracture with significant dorsal displacement and overlap of the fragments.

**Figure 2:**
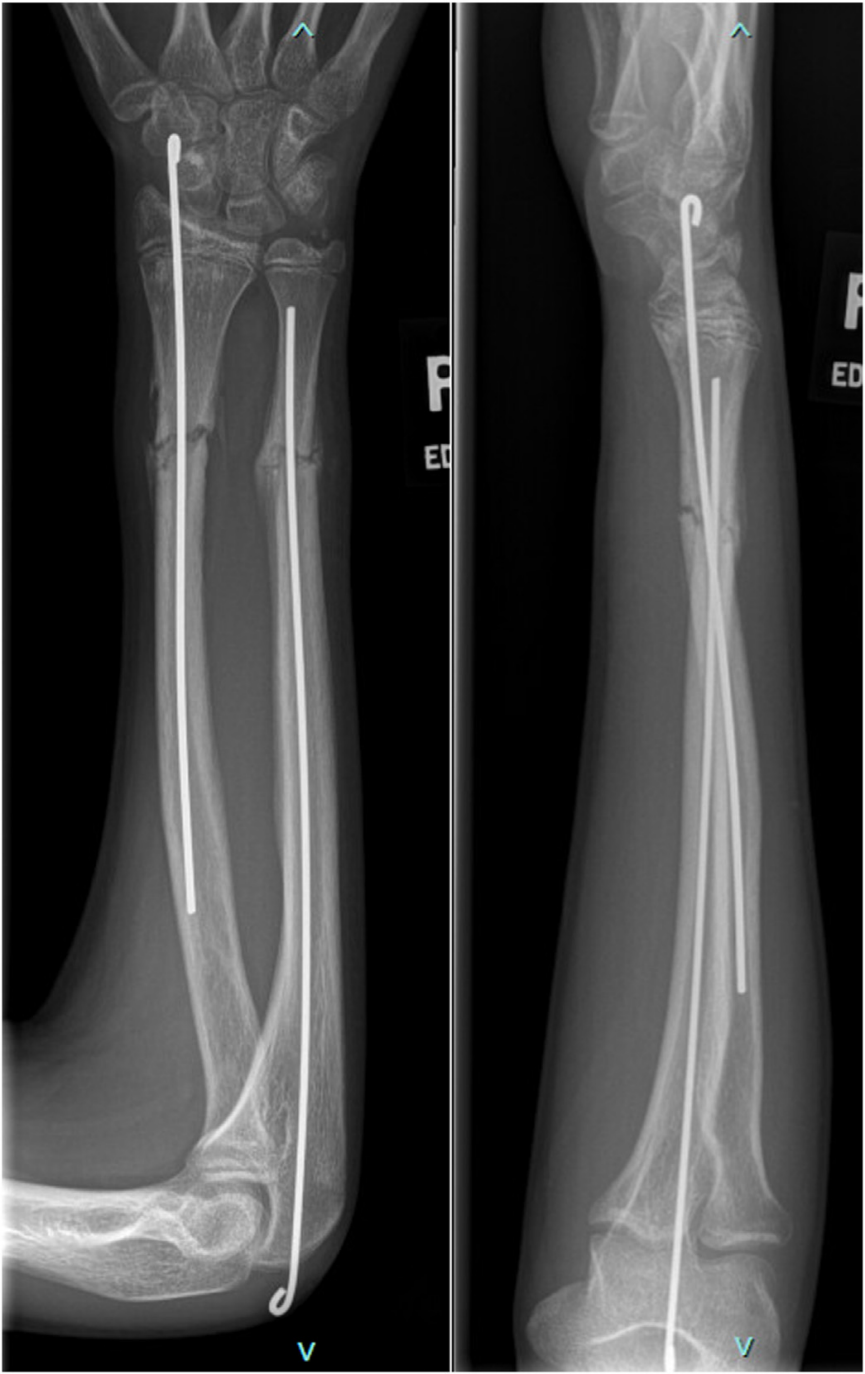
Lateral and poster–anterior radiographs taken after surgery demonstrating reduction of the fracture with flexible intramedullary nails.

**Figure 3:**
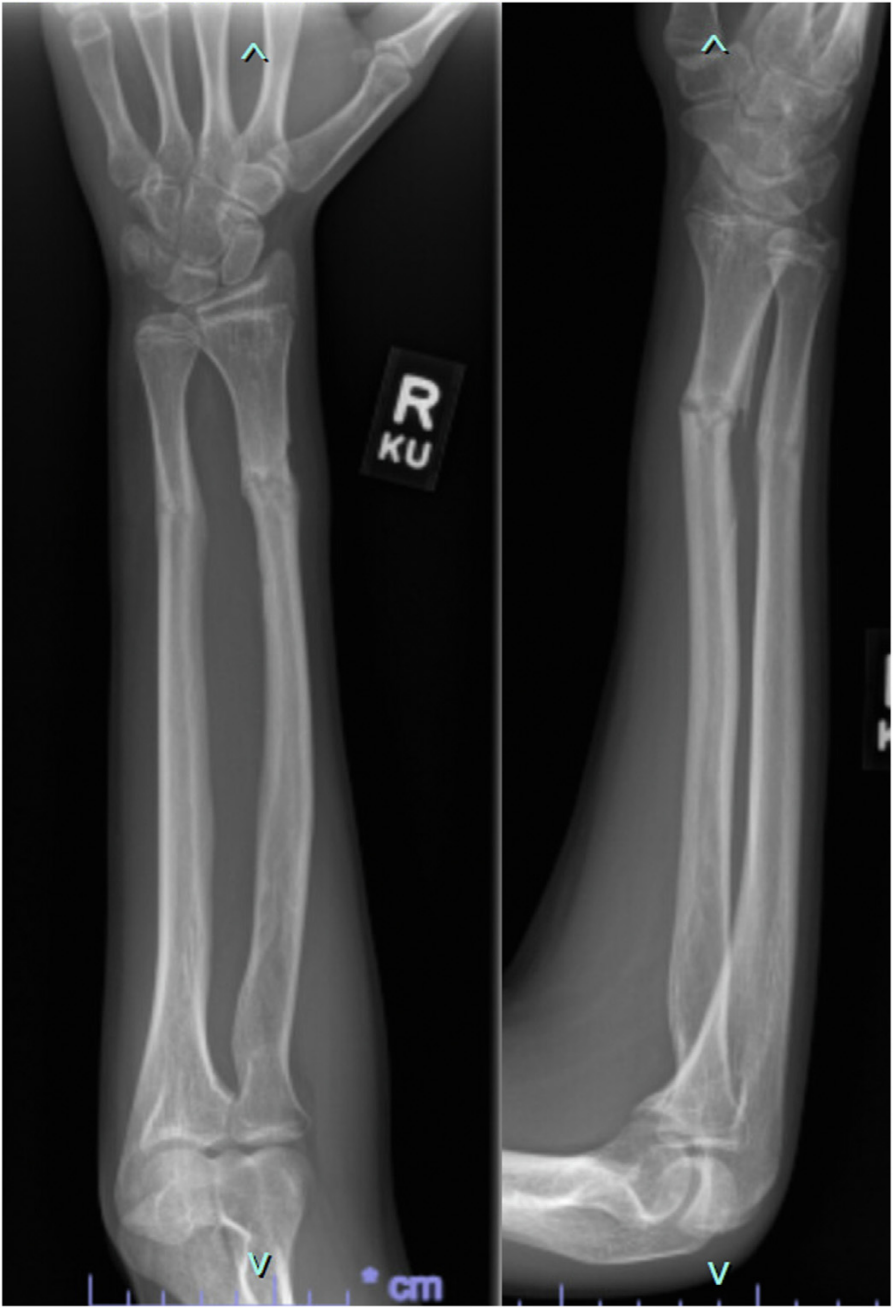
Lateral and poster–anterior radiographs taken 6 weeks after hardware removal demonstrating no evidence of abnormal fracture site lucency.

A 16-year-old boy presented to the emergency department after sustaining a Gustillo-Anderson type I open BBFFs after falling from his skateboard. His neurovascular examination was normal, and no deficits in finger or thumb flexion were noted. Orthogonal forearm x-rays revealed displaced distal radial and ulnar shaft fractures with notable overlap of the fracture fragments (Fig. 1). The patient underwent irrigation and debridement of the open fracture with flexible intramedullary fixation of the radial and ulnar fractures (Fig. 2). The patient was transitioned to a short arm cast for another 4 weeks. At 10 weeks after surgery, the patient returned to the operating room for hardware removal. Intraoperative evaluation revealed limited extension of the thumb interphalangeal (IP) joint even under anesthesia. At a 6-week follow-up visit, it was noted that the right thumb was held in a flexed position at 90° at the IP joint. X-rays obtained during this visit demonstrated partial healing of the radius and ulna without any visible lucencies or abnormal callus formation (Fig. 3).

The orthopedic team referred the patient to the hand clinic for concerns about a right thumb contracture 4.5 months after surgery. Upon presenting to our clinic, the patient endorsed forearm pain with an extension of the thumb. Examination demonstrated that the IP joint was held at 90° in flexion with attempted active extension but passively extended with the tenodesis effect associated with wrist flexion. There was no active flexion in either position (Video S1 available online on the Journal’s website at https://www.jhsgo.org). The patient was referred to hand therapy for tendon gliding and range of motion exercises, as the suspected diagnosis was scar adhesion of the FPL tendon. Ten weeks of hand therapy failed to provide any notable improvements. Given the suspected scar adhesions, the patient was then indicated for tenolysis versus tendon reconstruction of the FPL. The risks and benefits were discussed with the patient and his family, and informed consent was obtained to proceed with surgery.

The patient returned to the operating room 11 months after the initial injury. A standard Henry approach was performed. The FPL muscle belly and tendon were explored, which revealed that the tendon was wrapped around the shaft of the radius with the tendon partially encased in bone (Video S2 available online on the Journal’s website at https://www.jhsgo.org). Rongeurs were used to free the trapped tendon circumferentially. Given these findings, the options of radial osteotomy and tendon reduction versus tendon division, reduction, and repair were considered. Given that the tendon appeared to be of poor quality, the decision was made to proceed with division of the tendon with repair versus reconstruction. The tendon was divided and reduced to its anatomic position. However, the extracted ends were unsuitable for repair; hence, tendon reconstruction with palmaris longus (PL) autograft was performed. Ten centimeters of the patient’s PL tendon were harvested and secured with a Pulvertaft weave into the distal end of the FPL and secured with 4-0 nonabsorbable braided sutures at each juncture. The proximal end of the PL graft was secured similarly. After graft placement, tenodesis was checked, confirming an appropriate tensioning for the repair. The patient was placed into a plaster thumb spica splint. Two weeks after surgery, the patient was seen in the clinic for a wound check, splint change, and referral to hand therapy. Written informed consent was obtained from the patient to publish this case report and accompanying images. The patient missed several follow-up appointments until 5 months after surgery. At this point, his examination was consistent with his pre-op examination before undergoing tendon reconstruction. Specifically, the right thumb IP joint was held in 90° of flexion on extension but extended with wrist tenodesis. The importance of completing treatment and options moving forward were discussed with the patient, and another referral was placed to hand therapy. Unfortunately, the patient did not attend further follow-up appointments; hence, the final range of motion is not known. The patient has been contacted by phone and now reports no complaints about his thumb.

## Discussion

Pediatric BBFFs are rarely associated with flexor tendon entrapment (FTE). To date, there have been 19 cases of pediatric FTE following midshaft forearm fractures reported in the literature.^3^ Both bone forearm fractures–related entrapments have been characterized as having persistent cortical defects at the fracture site and flexion contracture that do not improve with conservative management.^3^ The FPL tendon has been involved in two prior cases—one with concomitant entrapment of the median nerve and another with localized myotendinous adhesions of the index finger FDP and FPL tendon at the radius fracture site.^2^^,^^4^ The patient presented is unique as the FPL was wrapped around the shaft of the radius, preventing him from straightening the thumb IP joint and requiring reconstruction of the FPL tendon.

Most FTEs have been documented as sequelae of proximal phalanx or distal radius fractures. Finger flexion deficits in the acute period can be caused by muscle belly within the fracture site or prolonged immobilization, whereas delayed presentations may be due to scar tissue or callus formation.^2^^,^^5^ Regardless of etiology, tendon entrapment is a rare complication that is often diagnosed late, with definitive management ranging from 2 days to 16 years.^3^^,^^5^ Delayed diagnoses stem likely result from failure to recognize the complication.^5^

The utility and timing of advanced imaging modalities are unclear. Magnetic resonance imaging is the gold standard for soft tissue injury; however, its use in children must be balanced with the need for another anesthesia event. Musculoskeletal ultrasound is sensitive to FTE; however, it requires an experienced operator and a cooperative patient.^5^ Computed tomography imaging has successfully identified FTE in adult patients.^5^ Although faster and more cost effective than magnetic resonance imaging, computed tomography imaging uses radiation, and cumulative doses of lifetime exposures should be considered.

We attempted a course of hand therapy for 3 months, given the most likely etiology for his inability to extend the thumb was scar adhesion. However, this failed to improve his symptoms. Although an earlier case report involving the FDP tendon suggests conservative management may be effective for early diagnosis of FTE, the success of such measures for our patient remains unclear. The location of the entrapment also drives treatment, with fracture cases involving the proximal one-third of the ulna being more amenable to conservative management.^5^ Fractures in the distal one-third are closer to the muscle-tendon junction, and surgical treatment may be required, which was ultimately the case for our patient.

The tendon grafts for deficient FPL include brachioradialis, flexor carpi radialis, extensor indicis propius, and PL, the latter used here.^6^ The PL remains one of the most used tendon autografts.^6^^,^^7^ The PL’s characteristics (length, diameter, availability, and accessibility) provided adequate length and strength while avoiding the need for an additional incision site.^7^ In pediatric patients, studies demonstrate no significant difference in grip strength between individuals with a PL and those without, supporting its use as a reasonable graft option.^8^ The alternative option, a radial osteotomy, is less frequently reported for tendon entrapment. One case report described a corrective osteotomy to address the interposition of the tibialis posterior tendon following an irreducible ankle fracture-dislocation.^9^ The osteotomy also corrected a medial malleolus malunion.^9^ Given the poor quality of the remaining FPL tendon, the decision was made to reconstruct the tendon rather than osteotomized the radius.

Previous case reports emphasize the importance of sufficient rehabilitation to restore tendon function. In an earlier case report with ring and little finger FTE, the patient regained full range of motion 5 years postmyotenolysis.^1^ Another FDP entrapment release resulted in full range of motion at 8 months following conservative management and extensive rehabilitation.^5^ Palmaris longus tendon grafts for FPL rupture secondary to open reduction and internal fixation with volar plate demonstrated varying levels of rehabilitation ranging from 6 months to almost 3 years, underscoring the lack of an optimal postoperative protocol and the need to tailor on a case-by-case basis. Moreover, early mobilization also depends on surgical technique. In zone five flexor tendon injuries lacking neurovascular involvement, early mobilization (as early as 1 day post-op) demonstrated low complication rates using a modified Kessler technique reinforced with a continuous running suture.^10^ Unfortunately, a limitation of our case is that our patient was inconsistent with postoperative follow-up visits and did not initiate hand therapy. Nonetheless, given the lack of improvement observed following entrapment release and reconstruction, it is challenging to anticipate the potential impact of aggressive hand therapy on the patient’s outcome, given the delay in recognizing the tendon entrapment early in treatment.

This is the first reported case of FPL tendon entrapment leading to translocation of the tendon dorsal to the radius following a pediatric BBFF requiring tendon division and reconstruction using PL autograft. When evaluating patients with similar injury mechanisms and presentations, hand surgeons must have a high index of suspicion for FTE in the fracture site. Intraoperatively, the risk of entrapment may be prevented by conducting a complete examination of passive motion and tenodesis after fracture fixation. Postoperative active range of motion examination in the post-anesthesia care unit should be performed to assess for any evidence of abnormal tendon gliding. Our case demonstrates a technique of surgical management for this uncommon complication.

## Conflicts of Interest

No benefits in any form have been received or will be received related directly to this article.

## Supplementary Data

Video S1 Preoperative video demonstrating the motion of the thumb IPJ.

Video S2 Intraoperative video demonstrating the dorsal transposition of the FPL tendon held in the forceps.
